# Correlational study on cyberbullying and social abilities in intercultural teenagers

**DOI:** 10.3389/fpsyg.2022.848678

**Published:** 2022-07-29

**Authors:** María Tomé-Fernández, José Manuel Ortiz-Marcos, Christian Fernández-Leyva

**Affiliations:** ^1^Department of Research Methods and Diagnosis in Education, Faculty of Education and Sports Sciences, University of Granada, Melilla, Spain; ^2^Department of Development and Educational Psychology, Faculty of Education and Sports Sciences, University of Granada, Melilla, Spain; ^3^Department of Research Methods and Diagnosis in Education, Faculty of Education Sciences, University of Granada, Granada, Spain

**Keywords:** cyberbullying, social abilities, teenagers, intercultural, correlational study

## Abstract

This article analyzes the relationship between cyberbullying profile by racist reasons and social abilities in a group of intercultural teenagers living in Spain (*N* = 1478). The study includes participants aged between 12 and 16 years old (*M* = 13.99; SD = 1.352). Of these, 738 were male (49.9%) and 740 were female (50.1%). A correlational study was carried out using online tools with suitable psychometrics parameters (content-construct validity and reliability). The first one was a scale that measured social abilities, and the second one evaluated racist or xenophobic cyberbullying, differentiating the victim and aggressor profiles. The results indicated five main findings: (1) generally, the participants analyzed present all their social abilities; (2) for the most part, these participants do not normally experience cyberbullying; (3) a positive correlation exists between the majority of social abilities analyzed and the cybervictim profile. It was also observed a negative correlation between the social ability associated with the ability of making requests and this profile; (4) there is a positive correlation among the six social abilities analyzed and the cyberaggressor profile; (5) the racist or xenophobic cyberbullying are driven not only by the absence of social abilities, but in some cases, they are also driven by socio-demographic variables (i.e., age and gender). Likewise, this work shows how the absence of some social abilities in some participants involve racist or xenophobic experiences as victims and as aggressors, which may be of interest for the analysis of teenagers’ behavior in intercultural contexts, as well as according to age and gender. More transcultural research need to be carried out to know the global perspective of the link between social abilities and the different profiles of racist and xenophobic cyberbullying, framed in the context of social psychology and studies of mass communication.

## Introduction

The massive use of the Internet characterizes the habitual forms of communication among adolescents (Hamilton et al., 2020; Schmidt and Kaess, 2020; Bowman-Smith et al., 2021; Sun et al., 2021), which facilitates the phenomenon of cyberbullying (Martin-Criado et al., 2021; Evangelio et al., 2022; Maftei et al., 2022). This type of harassment, like the traditional one, is usually based on rumors and seeks social exclusion and denigration of the victim, although it does not require physical proximity (Kliem et al., 2020). In addition, it presents its own characteristics, such as exposure to an infinite audience and the difficulty to discern the identity of the aggressor (Geng and Lei, 2021), causing damage to the mental state and social wellbeing of the victims (González-Cabrera et al., 2018; Fatumo et al., 2020), such as low self-esteem, poor academic performance, depression, social isolation, and suicide attempts (Hu et al., 2021).

Cyberbullying has become a common form of aggression among adolescents (Eronen et al., 2021; Islam et al., 2021; Pascual-Sánchez et al., 2021), where the aggressor enjoys absolute anonymity while carrying out risky behaviors, such as the disclosure of personal information, insults, or threats (Kwanya et al., 2021; Wang and Ge, 2021). In addition, according to various investigations (Cabrera et al., 2019; Betts et al., 2021; Pabian et al., 2021), victims tend to have little digital supervision from their parents, have low social support, and feel alone, which emphasizes and prolongs the silent suffering to which they are subjected (Runions et al., 2018). If these inconveniences are added to more specific difficulties of ethnic minorities, such as communication problems or discrepancies in values, attitudes, or traditions with a majority group (Cabrera et al., 2019), they convert adolescents from these minorities into recurrent groups for this type of bullying (Hong et al., 2021), experiencing what experts have called xenophobic and/or racist cyberbullying (Kilvington, 2020; Houkamau et al., 2021; Sánchez-Romero and Muñoz-Jiménez, 2021).

In this regard, several studies in European intercultural societies have identified adolescents from minority groups, such as Muslims, Asians, or those of African origin, among others, as a target group for racist or xenophobic cyberbullying (Espinoza and Wright, 2018; Yudes et al., 2020). According to Trieu and Lee (2017), the Asian race is considered a group vulnerable to cyberbullying; there are numerous negative prejudices toward their customs, traditions, and integration, since they are considered a financial risk for the host society. Regarding cybervictim profile, it is also revealed that there has been an increase in physical and cyber violent attitudes toward Muslims (Jones et al., 2018) after the 9/11 terrorist attacks. Similarly, other research concluded that people of African descent are vulnerable to cyberbullying in host countries where they are seen as groups of lesser value threatening finances, culture, national identity, or civil insecurity (Rodríguez-Hidalgo et al., 2018). These groups have in common being minorities in the populations analyzed (Blaya and Audrin, 2019), which highlights the need to detect this type of cyberbullying in adolescents of these groups for future social integration in adulthood (Yaman et al., 2019), for them to be part of democratic, plural, and inclusive societies (Kislyakov et al., 2021; Meter et al., 2021).

Xenophobic and/or racist cyberbullying is motivated by hatred toward different people due to their physical characteristics, religion, ethnicity, or language (Costello and Hawdon, 2018; Englander et al., 2018; Blaya and Audrin, 2019). Taking into account relevant research on the topic (Cabrera et al., 2019; Gutiérrez-Esparza et al., 2019; Keum and Cano, 2021), this type of harassment can be evidenced in three ways: as intercultural cyberbullying related to what Przybylski (2019) and Rudes and Fantuzzi (2021) warned by way of coercion on minorities; as racist threats promoted by fear of the unknown and different, which are usually linked to behavioral problems, such as name-calling or digital harassment (Murnion et al., 2018); and as identity usurpation, which is characterized by denigration, exclusion, and exposure of the victim’s privacy (Finkelhor et al., 2020).

Facing such a situation, it is necessary for adolescents to develop skills that support good use of digital media, in which respect and tolerance for oneself and for others are the fundamental premises (Bickham et al., 2021; Valdivia-Vizarreta et al., 2021; Ren et al., 2022). In this regard, different investigations (Nasaescu et al., 2018; Wang et al., 2019; Giumetti et al., 2021) have shown that the so-called social skills, when acquired, improve positive interpersonal relationships and prevent violent behaviors on the Internet. Since these users are characterized by being good communicators, they know how to negotiate conflicts in a constructive way, they seek help when they need it, and adopt responsible social behaviors (Del Toro and Wang, 2021; Przepiorka et al., 2021).

These skills are related to the ability to know how to say no and end interactions; with the expression of social rights; with the ability to defend their own rights; with expressing anger and disagreement; with making requests; and with the ability to know how to initiate interactions with the opposite gender (Cycyk and Hammer, 2018; Canabate et al., 2021; Hyun et al., 2021); its absence can lead to social isolation (Du et al., 2021; Osborn et al., 2021). Fundamental aspect by which its promotion in young immigrants or ethnic, cultural, or racial minorities is justified (Becker and Klein, 2021; Cadenas and Kiehne, 2021; Crooks et al., 2021) in which the academic and social integration is the end of actual education (Dennison and Geddes, 2021; Matejko et al., 2021; Sidler et al., 2021) based on the education of democratic values and a culture of peace.

In addition, it has been shown that, in ethnic, religious, and cultural minority groups, the absences of these skills are closely related to the appearance of depression, anxiety, and the decrease of self-control in adolescents (Lee et al., 2021; Przepiorka et al., 2021) that are reflected, in some cases, with hostile behaviors (Wasserman et al., 2021). Likewise, minority groups are less integrated into the environment, have lower levels of empathy, less self-control, greater impulsivity, and a higher index of antisocial behaviors, which makes it easier for them to assume the role of aggressor in racist or xenophobic cyberbullying (Zych and Llorent, 2021).

On the other hand, Rodríguez-Hidalgo et al. (2018) and Martínez et al. (2020) mention that the lack of social skills in intercultural contexts promotes hostile xenophobic behaviors (Peled, 2019), which enhances the intimidation of minority groups and their consequent defensive response as cyberaggressors (Alsawalqa, 2021). Being a cybervictim increases the risk of becoming a cyberbully (Quintana-Orts and Rey, 2018), as has been shown in various investigations (Baldry et al., 2015; Kim and Faith, 2019; Zych and Llorent, 2021), where they are often called cyberaggressors/cybervictim (Lee et al., 2018; Coyle et al., 2021).

Finally, it should be noted that there are studies that show that sociodemographic factors, such as age and gender, are influential in racist and/or xenophobic cyberbullying. As stated by Riaza and Rodriguez (2016); Brochado et al. (2017), and Chen et al. (2021), this type of harassment is common in ages between 12 and 16 years, since there is an excessive connection to mobile devices in daily life (Casado et al., 2019; Tynes et al., 2019; Cooney-O’Donoghue et al., 2021), which causes the transfer of traditional bullying behaviors to the online space (Noakes and Noakes, 2021). Additonally, related with previous researches, (Ferfolja et al., 2020; Shaw et al., 2020; Ullman, 2020), depending on gender, there are greater possibilities of being victims of stalkers (Francisco and Felmlee, 2021).

For example, Mena-Rodríguez and Velasco-Martínez (2017) and Mulholland et al. (2020) identified adolescents, especially of African and Islamic cultural origins (Butler-Barnes and Inniss-Thompson, 2020; Kowalski et al., 2020), as cybervictims. While the risk factors related to cyberaggressors are mainly linked to the male gender, which distorts the consequences of their own behaviors, blames the victims for their situation, has low levels of self-esteem, little empathy, and high levels of aggressiveness (Martinez-Ferrer et al., 2021; Oriol et al., 2021).

Taking into account the existing scientific literature, this study hypothesizes that there will be a positive correlation between few or no social skills and the fact of being victims or online aggressors for racist or xenophobic reasons. And the sociodemographic variables of age, gender, race, ethnicity, or religion influencing the cyberbullying of adolescents are analyzed (Leduc et al., 2018). Therefore, the research raises the following questions: How is the correlation between social skills and the profile of cyberaggressor or cybervictim for racist or xenophobic reasons of adolescents? How do the sociodemographic variables of age, gender, race, ethnicity, and religion influence the cyberbullying profiles analyzed?

To answer these questions, the study proposes the following objectives: To determine the correlation between social skills and the profiles of cybervictim and cyberaggressor for racist and xenophobic reasons of adolescents. And, on the other hand, to know the influence of the variables age, gender, race, ethnicity, and religión on the analyzed cyberbullying.

## Materials and methods

In this research, a correlational design is carried out, where the aim is to know the relationship among the six dimensions of the Social Skills Scale for Young Immigrants (SSSYI) (Tomé-Fernández et al., 2020) and the three categories of each of the subscales (cybervictims and cyberaggressors) that make up the Cyberbullying Scale for students with Cultural and Religious Diversity (CSCRD) (Tomé-Fernández et al., 2019). In addition to the influence of the variables age, gender, race, ethnicity, and religion on the analyzed cyberbullying.

### Participants

**FIGURE 1:**
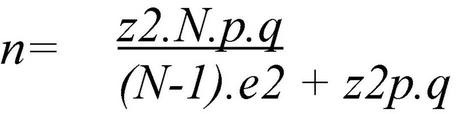
Statistical formula of the representative percentage of the sample. *n*, sample; *N*, population; *z*, 1.64; *p*, proportion (0.5); *q*, complementary proportion (0.5); *e*, standard error of estimation.

In this research, an intentional non-probability sampling was used. The number of participants analyzed (*N* = 1,478) in the study is acceptable. Since it exceeds the representative number of the total adolescent stipulated with the formula shown in Figure 1 (*n* = 386,821). This formula indicates a confidence level of 99% and a maximum estimation error of 5% (Yan et al., 2020). The sample was recruited in January 2021, and data collection took place between April 2021 and January 2022. In the collection of information, 10.75% of the data were lost, either because the legal guardians or parents did not give consent to participate in the study, or because they answered the questionnaires incorrectly.

The sample selection was made up of *N* = 1,478 adolescents from the provinces of Andalusia and the autonomous cities of Ceuta and Melilla. The participants are in the age range between 12 and 16 years (*M* = 13.99; SD = 1.352), in which a similar representation is reflected between 738 males (49.9%) and 740 females (50.1%).

In addition, 999 (67.6%) young people belong to the white race, 303 (20.5%) to the indigenous race, 66 (4.5%) to the black race, 21 (1.4%) to the Asian race, 14 (0.9%) to the Nordic race, and 75 participants (5.1%) did not answer this question.

On the other hand, 148 adolescents (10%) belong to the gypsy ethnic group, 7 (0.5%) to the Celtic, 17 (1.2%) to the Armenian, 99 (6.1%) to the Mongolian, 973 (65.8%) to the Castellana, and 234 (16.4%) did not answer this question.

Regarding the religion variable, 1,020 (69.0%) are Christian, 79 (5.3%) Hebrew, 60 (4.1%) Islamic, 16 (1.1%) Taoist, and 5 (0.3%) Buddhist. The rest of the participants, 298 (20.2%), did not practice any religion.

It is necessary to emphasize that belonging to a religion, ethnic group, or race is not an exclusive factor and may overlap. The southern provinces of Spain were chosen to carry out the study, as they are the Spanish areas where the greatest religious, cultural, and ethnic diversity is manifested in adolescents (García-Lastra and Sierra, 2021). The distribution of the participants by province is shown in Table 1.

**TABLE 1:** Distribution of participants according to the province.

Provinces	Frequency	%
Granada	403	27.3
Málaga	127	8.6
Almería	196	13.3
Jaén	65	4.4
Córdoba	80	5.4
Cádiz	204	13.8
Sevilla	78	5.3
Huelva	164	11.1
Melilla	81	5.5
Ceuta	80	5.4
Total	1478	100

### Instruments

Three instruments have been used in the study. The first one collected sociodemographic data, such as age, sex, province, nationality, institution, grade, race, ethnicity, and religion. The instrument has a reliability of α = 0.90.

The second instrument used was the SSSYI (Tomé-Fernández et al., 2020). The internal consistency indexes ranged between 0.81 and 0.89. This indicates that the instrument has a high level of reliability. For this, the construct validity and the reliability of the questionnaire were calculated using the Exploratory and Confirmatory Factor Analysis. Construct validity was analyzed using a data reduction technique. The extraction methods were Principal Component Analysis (PCA) and Varimax Rotation Method with Kaiser Mayer Olkin (KMO). First, the feasibility of performing this analysis was determined and confirmed by the values obtained by measuring the sample adequacy of KMO = 0.81 and Bartlett’s test of sphericity, *x*2 = 2344.36, *p* = 0.000. The analysis identified six factors that explained 43.09% of the variance. The distribution of items in six factors (the ability to say no and cut off interactions, self-expression in social situations, defense of rights, expression of anger or disagreement, the ability to make requests of others, and the ability to initiate interactions positive with people of the opposite sex) coincides with the hierarchical structure of the reference instrument (Mendo-Lázaro et al., 2016), which bases the dimensions of social skills on important theoretical references (Buchanan et al., 2016; Espinosa et al., 2016; Grabowska, 2016; Menesini and Salmivalli, 2017; Baton et al., 2019; In et al., 2019). The eigenvalues (amount of variance in the original set of variables explained by each principal component) greater than 1 and the factor load greater than 0.30 were established *a priori* (Siembida et al., 2018) as acceptable criteria for this analysis. This led to the decision to eliminate those items that saturated below 0.30 in the factor that theoretically should do it (items 7, 9, 14, and 16), moving the questionnaire from the initial 33 items to the final 29. The reliability of the questionnaire was calculated using Cronbach’s alpha internal consistency index. The total value was α = 0.89. Finally, the confirmatory factor analysis was performed, which showed excellent values (χ^2^ = 765.98, *p* = 0.00, NNFI = 0.82, CFI = 0.88, TLI = 0.85, IFI = 0, 83, and RMSEA = 0.04) (Machimbarrena et al., 2021; Corcoran and O’Flaherty, 2022).

The third instrument used was the Cyberbullying Scale for Students with Cultural and Religious Diversity (CSCRD) (Tomé-Fernández et al., 2019). In this questionnaire, the construct validity and its reliability were calculated through the Exploratory and Confirmatory Factor Analysis. Construct validity was analyzed using a data reduction technique. The extraction methods were PCA and Varimax Rotation Method with KMO. First, the feasibility of performing this analysis was determined and confirmed by the values obtained by measuring the sample adequacy of KMO = 0.926, and Bartlett’s test of sphericity, *x*2 = 31491.842, *p* = 0.000. The analysis identified three factors in each subscale (cybervictims and cyberaggressors) that represent 69.42% of the total variance explained, with saturation loads ranging between 0.312 and 0.904. The distribution of the items of each subscale (intercultural cyberbullying, digital racist threats, and usurpation of identity to people of different ethnicities, cultures, and religions) coincides with the hierarchical structure of the reference instruments (Del Rey et al., 2015; Hall, 2016; Romera et al., 2016; Elipe et al., 2017). Values greater than 1 and factor load greater than 0.30 were established *a priori* (Daryoushi et al., 2021) as acceptable criteria for this analysis. After analyzing the variance eigenvalues for each main component and obtaining a factor load greater than 0.30, the questionnaire was maintained with the 38 initial items related to cyberaggression and cyberbullying. The reliability of the questionnaire was calculated using Cronbach’s alpha internal consistency index. The total value for this scale was α = 0.90, obtaining excellent results (Correa and Sánchez, 2018). On the other hand, the confirmatory factor analysis showed an optimal fit to the model (χ^2^ = 2414.536, *p* = 0.00, NNFI = 0.80, CFI = 0.83, TLI = 0.81, IFI = 0.80, and RMSEA = 0.05) (Corcoran and O’Flaherty, 2022).

### Procedure and information gathering

For data collection, access to public compulsory secondary schools in the different provinces of southern Spain was gained. These centers were selected because of the large number of intercultural students. First of all, the participation of those responsible for the educational centers was requested by e-mail and telephone calls. Next, parents and legal guardians were asked to sign a consent form for the adolescents’ participation in the study, of which 124 (8.39%) did not sign. In this form, it was explained that participation was completely anonymous and that data confidentiality would be guaranteed.

Regarding the administration of the questionnaire, it was delivered in Spanish and virtually through the Google Forms platform to the adolescents. Previously, the teacher explained to them that they had to complete the questionnaire by filling in the answers with an x and that they could only select one answer from the different options provided in the two instruments: CSCRD (Tomé-Fernández et al., 2019), this instrument has two parts, the first part assesses the sociodemographic variables of age, gender, city, institution, course, nationality, ethnicity, culture, and religion; and the second part evaluates the cybervictim and cyberbully profile of the subjects surveyed. This part is a Likert scale made up of 38 items with five response options, ranging from never (1) to always (5). And the SSSYI is an instrument that is structured in two parts; on the one hand sociodemographic variables, such as age, sex, province, and nationality. And, on the other hand, it consists of a 29-item Likert scale with four response options ranging from never (1) to always (4) (Tomé-Fernández et al., 2020). The application of both questionnaires had an average duration of between 20 and 40 min.

Throughout this data collection procedure, the ethical recommendations provided in the Declaration of Helsinki (1975) were followed, later updated in Brazil in 2013 and by the Ethics Committee of the University of Granada (reference code: 742/CEIH/2018).

### Statistical analysis of the data

For the statistical analysis, the IBM SPSS Statistics 25 software was used. When the psychometric characteristics of the instruments used were guaranteed, it was determined whether the data complied with a normal distribution. The objective was to make decisions about using parametric or non-parametric tests. For this, the Kolmogorov–Smirnov test was used on the data collected. The results were significant (*p* < 0.05), so the null hypothesis about the Gaussian distribution of the data was rejected. Likewise, it was evaluated whether the requirement of homoscedasticity or equality of variances between the comparison groups was met. When detecting that the distribution of the data was not normal, one can speak of heteroscedasticity, because the error variance of the variables was not constant. Based on the above, non-parametric tests were performed. In this sense, the Spearman correlation test was used to analyze the relationship between the six categories of the SSYI and the three factors of each subscale of the CSCRD.

To determine whether there were significant differences according to gender, the Mann–Whitney *U* test was used, while the Kruskal–Wallis *H* test was used for the analyses carried out according to age, race, ethnicity, and religion. Finally, a hierarchical regression analysis was performed to determine the incremental validity of the sociodemographic variables analyzed (age, gender, race, ethnicity, and religion).

## Results

### Descriptive analysis

To find out to what extent the adolescents analyzed had social skills or had experienced cyberbullying, as aggressors or victims, the percentages of the responses given to the two scales were calculated. As indicated in Table 2, in the SSSYI, the vast majority of the adolescents analyzed present the six types of social skills evaluated. With a higher percentage (95.5%), the social ability is called “self-expression in social situations” and the lowest percentage (84.6%) corresponds to the social ability “make requests to others.”

**TABLE 2:** Percentage of responses given in the social skills scale for young immigrants (SSSYI) and cyberbullying scale for students with cultural and religious diversity (CSCRD).

	Percentages in %					
**Dimensions SSSYI**	**F1**	**F2**	**F3**	**F4**	**F5**	**F6**
	90.1	95.5	90.3	90.1	84.6	89.1
**Dimensions CSCRD**	**F1**	**F2**	**F3**			
Cybervictims	3.8	8.5	8.1			
Cyberbullies	2.5	4.8	6.4			

SSSYI (F1, say no and cut off interactions; F2, self-expression in social situations; F3, defense of rights; F4, expression of anger or disagreement; F5, make requests to others; F6, initiate positive interactions with people of the opposite sex). CSCRD (F1, intercultural cyberbullying; F2, digital racist threats; F3, usurpation of identity to people of different ethnicities, cultures, and religions).

On the other hand, the percentages obtained for the CSCRD indicate that most of these adolescents do not tend to experience cyberbullying, being only 20.4% of those who acknowledge having lived some experience of cyberbullying as victims and 13.7% of those who indicate that they have been aggressors. On this scale, the victims have scored a higher percentage, with scores similar to the dimensions “racist digital threats” (8.5%) and “identity theft from people of different ethnicities, cultures, and religions” (8.1%). This last dimension stands out in the profile of cyberbullies, since they also report suffering this type of harassment (6.4%).

### Correlation between the dimensions of the social skills scale for young immigrants and cyberbullying scale for students with cultural and religious diversity

To know the relationship among the six dimensions of the SSSYI and the three dimensions of each of the subscales (cybervictims and cyberaggressors) that make up the CSCDR, an analysis corresponding to the Spearman regression was carried out (Table 3).

**TABLE 3:** RHO Spearman (RHO) Spearman for social skills according to cybervictims and cyberbullies profiles.

Cybervictims	SSSYI						
		**F1**	**F2**	**F3**	**F4**	**F5**	**F6**
CSCRD	**F1**	0.778	0.746	0.716	0.743	-0.203	0.717
	**F2**	0.794	0.747	0.721	0.745	-0.208	0.724
	**F3**	0.807	0.761	0.731	0.751	-0.217	0.737

**Cyberbullies**	**SSSYI**						

		**F1**	**F2**	**F3**	**F4**	**F5**	**F6**
CSCRD	**F1**	0.699	0.698	0.548	0.608	0.196	0.508
	**F2**	0.705	0.702	0.556	0.610	0.202	0.507
	**F3**	0.712	0.706	0.571	0.626	0.201	0.513

All correlations have a sig. *p* = 0.000.

Regarding the cybervictim profile, the data indicate that most of the correlations between the dimensions of the SSSYI and the cyber victim subscale of the CSCDR are high and positive. Only negative correlations are observed between the dimension “Make requests to others” (F5 of the SSSYI) and the three factors of the cyber victim subscale.

Regarding the cyberbully profile, all the correlations obtained are high and positive.

In both profiles, the highest correlations were between the dimensions “Say no and cut interactions” (F1 of the SSSYI) and “Usurpation of identity to people of different ethnicities, cultures, and religions” (F3 of the CSCDR), with an *r* = 0.807 in the case of the victims and with an *r* = 0.712 in that of the aggressors.

### Profiles according to age, gender, ethnicity, race, and religion

To determine if the age variable influences the two subscales of the CSCRD, the Kruskal–Wallis *H*-test was performed for the age ranges between 12 and 14 years and 15 and 16 years (Table 4).

**TABLE 4:** Kruskal–Wallis *H*-test for cybervictims and cyberbullies according to the age range.

Years	Cybervictims		
	**F1**	**F2**	**F3**
12–14	1.128	1.341	1.196
15–16	0.402	0.163	0.117

**Years**	**Cyberbullies**		

	**F1**	**F2**	**F3**
12–14	5.819	6.226	5.771
15–16	1.731	1.921	1.109

The youngest age group (12–14 years old) turned out to be the most likely to experience racist or xenophobic cyberbullying as victims and as aggressors, highlighting that in both profiles, the dimension “Digital racist threats” (F2 of the CSCRD) with a *x*2 = 1,341 for the victims and a *x*2 = 6,226 for the aggressors, at a significance level of *p* = 0.000. To determine the behavior of racist or xenophobic cyberbullying according to gender, the Mann–Whitney *U*-test was carried out in each of the CSCRD subscales (Table 5).

**TABLE 5:** Mann–Whitney *U* for cybervictims and cyberbullies according to gender.

Gender	Cybervictims		
	**F1**	**F2**	**F3**
Male	69615.500	67431.000	66468.500
Female	70515.500	69935.500	69870.000

**Gender**	**Cyberbullies**		

	**F1**	**F2**	**F3**
Male	67738.500	67618.500	65803.500
Female	65286.000	65069.000	65610.000

The results indicate that female adolescents are the most likely to be cybervictims in the three dimensions analyzed: “Intercultural cyberbullying” with *U* = 70515.500, “Digital racist threats” with *U* = 69935.500, and “Usurpation of identity to people of different ethnicities, cultures, and religions” with *U* = 70515.500.

Regarding the male gender, the data present them as cyberaggressors in this type of cyberbullying in the three dimensions analyzed: “Intercultural cyberbullying” with *U* = 67738.500, “Digital racist threats” with *U* = 67618.500, and “Usurpation of identity to people of different ethnicities, cultures, and religions” with *U* = 65803.500.

Next, to determine the influence of the race, ethnicity, and religion variables, the Kruskal–Wallis *H*-test was performed (Tables 6–8).

**TABLE 6:** Kruskal–Wallis *H*-test for cybervictims and cyberbullies according to race.

Race	Cybervictims					
	**F1**	**IC 95%**	**F2**	**IC 95%**	**F3**	**IC 95%**
African	1.457	75–76	1.953	82–84	1.754	73–75
Asian	3.803	69–71	3.590	67–69	3.762	69–72
Indigenous	0.487	70–72	0.468	65–67	0.423	73–75
White	0.396	80–82	0.308	81–83	0.454	77–79
Nordic	0.487	70–72	0.358	71–73	0.389	68–70
Others	0.332	69–71	0.357	66–68	0.302	64–66

**Race**	**Cyberbullies**					

	**F1**	**IC 95%**	**F2**	**IC 95%**	**F3**	**IC 95%**
African	1.354	73–75	1.791	75–77	1.344	69–71
Asian	0.544	65–67	0.680	73–75	0.766	67–69
Indigenous	0.343	81–83	0.451	79–81	0.543	67–69
White	3.828	64–66	3.438	59–61	3.532	65–67
Nordic	0.334	59–61	0.421	76–78	0.462	81–83
Others	0.321	56–58	0.295	59–61	0.264	54–56

**TABLE 7:** Kruskal–Wallis *H*-test for cybervictims and cyberbullies according to ethnicity.

Ethnicity	Cybervictims					
	**F1**	**IC 95%**	**F2**	**IC 95%**	**F3**	**IC 95%**
Gypsy	3.423	80–92	3.653	90–0	2.987	81–92
Celtic	0.432	65–69	0.321	62–65	0.385	67–69
Armenia	0.267	60–64	0.643	59–60	0.453	67–69
Mongolian	0.631	64–66	0.543	64–66	0.639	65–67
Castilian	0.448	56–62	0.637	57–61	0.538	81–83
Others	0.375	58–62	0.319	50–62	0.204	54–56

**Ethnicity**	**Cyberbullies**					

	**F1**	**IC 95%**	**F2**	**IC 95%**	**F3**	**IC 95%**
Gypsy	1.325	69–71	1.763	69–71	1.032	69–71
Celtic	0.304	67–69	0.418	67–69	0.387	67–69
Armenia	0.393	67–69	0.451	67–69	0.480	60–71
Mongolian	0.402	65–67	0.441	65–67	0.427	64–67
Castilian	0.308	81–83	0.456	81–83	0.421	88–86
Others	0.276	54–56	0.318	54–56	0.475	55–62

**TABLE 8:** Kruskal–Wallis *H*-test for cybervictims and cyberbullies according to religion.

Religion	Cybervictims					
	**F1**	**IC 95%**	**F2**	**IC 95%**	**F3**	**IC 95%**
Judaism	1.123	69–71	1.943	69–71	1.407	70–77
Christianity	0.603	67–69	0.576	67–69	0.322	50–66
Islam	2.367	67–69	4.664	67–69	3.753	80–65
Buddhism	0.654	65–67	0.960	65–67	0.673	65–67
Taoism	0.438	81–83	0.238	81–83	0.302	81–83
Others	0.327	54–56	0.212	54–56	0.289	54–56

**Religion**	**Cyberbullies**					

	**F1**	**IC 95%**	**F2**	**IC 95%**	**F3**	**IC 95%**
Judaism	1.129	70–75	0.798	69–71	1.774	69–71
Christianity	0.504	57–68	0.480	52–67	0.456	70–81
Islam	4.797	70–75	5.421	70–78	4.210	56–80
Buddhism	0.930	80–68	0.648	65–77	0.439	65–67
Taoism	0.394	85–60	0.643	81–83	0.587	87–83
Others	0.210	40–58	0.391	54–57	0.342	54–56

The findings in Table 6 show how Asian adolescents are the most likely to suffer from racist or xenophobic cyberbullying, obtaining the tendency to be cyberaggressors to white adolescents. In both profiles, the dimension “Intercultural cyberbullying” (F1 of the CSCRD) stands out with a *x*2 = 3,803 for Asian victims and a *x*2 = 3,828 for white aggressors, at a significance level of *p* = 0.000.

For the ethnic variable, the gypsy group was the most likely to experience racist or xenophobic cyberbullying, both as victims and aggressors. Highlighting in both profiles the dimension “Digital racist threats” (F2 of the CSCRD) with a *x*2 = 3.653 for the victims and a *x*2 = 1.763 for the aggressors, at a significance level of *p* = 0.000.

The results shown in Table 8 show that adolescents of the Islamic religion are the most prone to experience racist or xenophobic cyberbullying in the role of the victims and that of the aggressors, highlighting that in both profiles the dimension “Digital racist threats” (F2 of the CSCRD) with a *x*2 = 4,664 for the victims and a *x*2 = 5,421 for the aggressors, at a significance level of *p* = 0.000.

### Incremental validity of sociodemographic variables

To determine to what extent the three factors of the two subscales (cybervictim and cyberaggressor) of the CSCRD were driven by sociodemographic variables (age, gender, race, ethnicity, and religion) and by the social skills analyzed in the SSSYI, a series of stepwise linear regressions was analyzed. These analyses shed light on how much variance of the dimensions of the CSCRD are explained by the sociodemographic variables and by the social skills evaluated through the SSSYI. In this line, the results shown in Table 9 indicate that the analyzed variables represent significant proportions of variance for each of the dimensions of the two subscales that make up the CSCRD. With adjusted *R*^2^ values ranging from -0.002 for gender to 0.983 for social skills in the cybervictim profile and from 0.005 for gender to 0.976 for social skills in the cyberaggressor profile (Table 9).

**TABLE 9:** Linear regressions for the variables social skills, age, gender, race, ethnicity, and religion.

*R*^2^ adjusted	Social skills	Age	Gender	Race	Ethnicity	Religion
**Cybervictims**						
CSCRD	0.983	0.021	-0.002	0.018	0.022	0.015
**Cyberbullies**						
CSCRD	0.976	0.006	0.005	0.007	0.009	0.006

All correlations have sig. *p* = 0.000.

## Discussion

This research focused, in the first place, on verifying if the participants presented the social skills defined in the SSSYI scale, and if they were participants in racist or xenophobic experiences of cyberbullying, which is evaluated through the CSCRD. The data obtained through the percentages of the answers show that the adolescents evaluated have, for the most part, social skills and that, in general, they had no experiences related to this type of cyberbullying (Tanrikulu and Erdur-Baker, 2019). This is consistent with previous studies, which indicate that having social skills is less frequent to become involved as a victim or stalker in situations of cyber violence (Bradbury et al., 2018; Acosta et al., 2019; Yang et al., 2021). Subsequently, the Spearman correlation test was applied to analyze the relationship between the six categories of the SSYI scale and the three factors of each subscale of the CSCRD. In line with previous studies (Bradbury et al., 2018; Rodríguez-Álvarez et al., 2021), the data indicated that the two subscales of the CSCRD are positively correlated with most of the social skills analyzed. However, attending to the cybervictim profile, negative correlations were observed with the social skill related to the fifth dimension (the ability to make requests to others) of the Social Ability Scale for Young Immigrants (SSYI) scale. This implies that in this type of harassment, as shown in previous research on traditional or cyberbullying (Wachs et al., 2019; Ybarra et al., 2019), the fact of making requests, such as asking to stop the threat or insult, does not imply that this happens, but rather that in some cases it increases the intensity and frequency of the harassment.

In addition, the results obtained through the Spearman correlation also indicated that in both profiles (cybervictim and cyberaggressor), the highest correlation was between the dimensions “Say no and cut interactions” and “Usurpation of identity to people of different ethnicities, cultures, and religions.” This is in agreement with another study, where those subjects who knew how to set limits in their interactions were the least likely to suffer bullying and to generate it (White and Crandall, 2017). This may be due to the fact that these people will present less favor toward the prototype of the bully, they will justify themselves less, they will not accept denigration, exclusion, the usurpation of privacy, and they will feel less guilty when saying no and cutting off negative interactions; these aspects are highlighted by Zych et al. (2015) and by Yudes et al. (2020) as psychological and individual risk factors in victims.

These findings are also consistent with evidence demonstrated in studies on racist or xenophobic cyberbullying (Giumetti et al., 2021; Guo, 2021). Along these lines, authors such as Peled (2019) argue that the lack of skills to exercise good intercultural communication makes them more vulnerable to being bullied. Rodríguez-Hidalgo et al. (2018) indicate that those with limited skills to manage multicultural interactions in situations of anger and frustration show fewer resources to resolve interpersonal conflicts and resort more to aggression as a means of problem solving (Coelho and Marchante, 2018).

On the other hand, the fact that this research shows a greater propensity to be cybervictims in adolescents of the age range between 12 and 13 years agrees with previous studies where this priority rank is obtained in the role of victims in face-to-face harassment situations (Gómez et al., 2020; Özbey and Başdaş, 2020). This may be due to the fact that it is the age at which adolescents are most active on the Internet, thereby increasing digital interactions that sometimes involve cyberbullying (Selkie et al., 2016; Perret et al., 2020). In addition, the decrease in racist or xenophobic cyberbullying in older adolescents confirms the conclusion of previous research which showed that racism or xenophobia decreased as age increased (Giménez et al., 2015; Sittichai and Smith, 2018). In the case of cyberbullying, this may be due, according to Resnicow (2019), to the fact that younger adolescents present great impulsiveness in the management of social networks, using them with low levels of assertiveness and social skills, and with deficiencies that improve to as they grow and control themselves. This indicates the gradual disappearance of cyberbullying in older samples (Jenaro et al., 2017; Savage and Tokunaga, 2017; Alipan et al., 2018; DeSmet et al., 2018).

In addition, the results obtained highlight this same age range as the most prone to being cyberaggressors, thus coinciding with a previous study (Machimbarrena and Garaigordobil, 2017). Positioning the age range between 12 and 13 years as the stage in which adolescents are more likely to be cyber victims and xenophobic or racist cyberbaggressors. This can be explained according to Tran et al. (2021) that victims in stressful situations can acquire different coping strategies in which they become an aggressor as revenge.

Likewise, the findings obtained in this research regarding gender show that this variable influences bullying produced through digital media. More specifically, in the study, it was found that female adolescents stand out as cyber victims, while the male gender is related to the cyberaggressor profile (Pacheco-Salazar and López-Yáñez, 2019; Celuch et al., 2021). These data coincide with the socially stipulated stereotypes, ratified in previous studies (Donoso-Vázquez et al., 2017; Jiménez, 2019). The data obtained from the INE (2020) indicate that adolescents are the ones with the highest percentage of online bullying (65.44%), of which 25.7% are related to racist or xenophobic bullying. In this regard, it is highlighted that female racial discrimination is considered a potentially motivating variable for cyberbullying (Dambrun et al., 2016; Watson et al., 2020). Previous research has shown that (Chen and Cheng, 2017; Choi and Lee, 2017; Khalfaoui et al., 2020), where gender and cultural inequality has been showed through the network. Even online harassment crimes produced in Spain involve a greater proportion of the male gender (INE, 2020). This highlights the need to better understand the context in which it occurs to reduce negative interactions and aggressions (Burger and Bachmann, 2021).

On the other hand, the results obtained with respect to the variables race, ethnicity, and religion show that adolescents of the Asian race are the most likely to be cybervictims for racist or xenophobic reasons. This, as indicated by Barlett and Wright (2017) may be due to the fact that, at present, this group has become a focus of attention in social networks, where they are blamed for the spread of the COVID-19 virus, and where they are sometimes ridicule and mock in a dehumanized and insulting way (Rao et al., 2019). On the other hand, the white race is identified as the most prone to being a cyberaggressor. In the context where the research is developed, this breed is the dominant one (Wang and Ngai, 2021). Aspect that conditions conflicts promoted by power struggles, where majorities tend to be violent toward minority groups who believe that they put cultural or identity traditions at risk (Özdemir et al., 2018; Blaya and Audrin, 2019).

Regarding the ethnicity variable, the results show that the gypsy ethnic group is the most likely to be cybervictims and cyberaggressors in this type of cyberbullying, which also agrees with other studies (Atak, 2021; Sánchez-Romero and Muñoz-Jiménez, 2021). Furthermore, Carcelen and Martinez (2021) and Kim et al. (2021) state that students of gypsy origin are often rejected and hated not only on the Internet, but also in everyday contexts. This may be due to the fact that they constitute a minority group that throughout history has been perceived as violent and marginal in the Spanish population (Rosário et al., 2020). On the other hand, the data also identify adolescents of the Islamic religion who tend to be cybervictims and cyberaggressors. In this regard, there are investigations (Lapidot-Lefler and Hosri, 2016; Schmuck et al., 2017) that detect an increase in violent attitudes toward this group, reinforced by the terrorist attacks that have occurred in recent decades and by fear to the radicalization of this collective (Oksanen et al., 2018; Ayala et al., 2019).

Both in the gypsy ethnic group and the Islamic religion, the results have detected that they are prone to being cybervictims and cyberaggressors in this type of cyberbullying. A phenomenon that may be due to the defensive response that these groups have when feeling threatened (Bai et al., 2020; Alsawalqa, 2021). Since, as indicated by Lee et al. (2018) and Quintana-Orts and Rey (2018), being a cybervictim increases the risk of becoming a cybercriminal, these groups are called cyberaggressor/cybervictim (Baldry et al., 2015; Kim and Faith, 2019; Zych and Llorent, 2021). These results ratify that the cultural background of adolescents is a crucial factor for social and educational research that will determine future practice and preventive programs (Hecker et al., 2018; Celuch et al., 2022).

Finally, it is worth mentioning that the factors that influence cyberbullying have been studied intensively since the 21st century (Harrison and Polizzi, 2021); however, there are still some gaps in the knowledge of racist or xenophobic cyberbullying (Albdour et al., 2019). Since most of the studies that focus on this topic have been done without taking into account possible differences among groups based on predictor variables (Festl et al., 2017; Zych and Llorent, 2021), which makes this research a relevant study on the subject (Murnion et al., 2018) by delving into the existing correlations with social skills, as well as with age, gender, race, ethnicity, and religion. These are aspects of great importance in adolescence, as it is a critical period in the transition to adult life, where they are vulnerable to external influences and are characterized by immaturity and lack of self-control (Hutson et al., 2018; Kaltenbach et al., 2018).

## Conclusion

In general, the adolescents analyzed present all social skills. Despite this, the skill that appears in a lower percentage is that related to “Making requests to others” and to a greater extent related to the ability to “Self-expression in social situations.”

Most of the participants have stated that they have not had experiences related to racist or xenophobic cyberbullying. Only 20.4% have suffered it as victims, and 13.7% have carried it out as aggressors.

On the other hand, there are positive correlations between most of the social skills analyzed and the dimensions that make up the cybervictim profile. Only negative correlations are observed between social ability linked to the ability to make requests and the factors of this profile. This shows that the deficiency in making requests to others, in some participants, has led them to live situations where they usurped their identity or threatened or/and insulted them for racist or xenophobic reasons.

There is also a positive correlation among the six social skills analyzed and the cyberaggressor profile. This shows that the absence of social skills related to saying no and cutting off interactions, self-expression in social situations, the defense of rights, expression of anger or disagreement, making requests to others, and initiating positive interactions with people of the opposite sex can lead the adolescent suffering racist or xenophobic cyberbullying or generating it.

Finally, it is worth mentioning that this type of cyberbullying is driven not only by the absence of social skills, but also by sociodemographic variables, such as age, gender, race, ethnicity, and religion. In this regard, it has been shown that female adolescents aged between 12 and 13 are the most likely to be cybervictims for racial or xenophobic reasons. In addition, the Asian race is identified as the most likely to be cybervictims of this type of cyberbullying, while the white race is the most likely to be cyberaggressors.

On the other hand, regarding ethnicity and religion, the gypsy and the Islamic, respectively, stand out as the most likely to be cybervictims and cyberaggressors.

The results obtained lay the foundations for important practical implications. Detecting the positive correlation between social skills and cyberbullying can help psychoeducational professionals create preventive interventions aimed at reinforcing and acquiring the aforementioned skills. In addition, knowing the profiles most likely to be cybervictims or cyberaggressors will allow specializing interventions with attractive didactic materials in line with the cultures that most need them.

## Limitations

In this research, although the sample selection is representative, according to the statistical calculations established by Vanderplas et al. (2020), the sample selection was made only in the south of Spain; some cultural minorities more typical of the center or north of the country, such as the Buddhist religion or the Celtic ethnic group, have been left out of the study. This means that the results obtained are biased and do not represent the Spanish or European territory. That is why it would be convenient to carry out more studies of this type in other geographical areas to know the global perspective of the link between social skills and the different profiles of racist or xenophobic cyberbullying, contextualized in social psychology and research on digital media. Even so, the study fills an existing research gap on the topic, since it correlates social skills and different profiles of cyberbullying in the Spanish intercultural context for the first time.

## Data availability statement

The original contributions presented in this study are included in the article/supplementary material, further inquiries can be directed to the corresponding author.

## Ethics statement

Throughout this data collection procedure, the ethical recommendations provided in the Declaration of Helsinki (1975) were followed, later updated in Brazil in 2013 and by the Ethics Committee of the University of Granada (reference code: 742/CEIH/2018). Written informed consent to participate in this study was provided by the participants or their legal guardian/next of kin.

## Author contributions

JO-M, MT-F, and CF-L conceived the hypothesis of this study. JO-M participated in data collection. All authors analyzed the data, contributed to data interpretation of the statistical analysis and wrote the manuscript with significant input, and read and agreed to the published version of the manuscript.
